# Applying post-neoadjuvant pathologic stage as prognostic tool in esophageal squamous cell carcinoma

**DOI:** 10.3389/fonc.2022.998238

**Published:** 2022-11-10

**Authors:** Weiming Han, Wei Deng, Qifeng Wang, Wenjie Ni, Chen Li, Zongmei Zhou, Jun Liang, Dongfu Chen, Qinfu Feng, Nan Bi, Tao Zhang, Xin Wang, Lei Deng, Wenqing Wang, Wenyang Liu, Jianyang Wang, Qi Xue, Yousheng Mao, Xiangyang Liu, Dekang Fang, Jian Li, Dali Wang, Jun Zhao, Zefen Xiao

**Affiliations:** ^1^ Department of Radiation Oncology, National Cancer Center/National Clinical Research Center for Cancer/Cancer Hospital, Chinese Academy of Medical Sciences and Peking Union Medical College, Beijing, China; ^2^ Department of Radiation Oncology, Peking University School of Oncology, Beijing Cancer Hospital and Beijing Institute for Cancer Research, Beijing, China; ^3^ Radiation Oncology Key Laboratory of Sichuan Province, Department of Radiation Oncology, Sichuan Cancer Hospital and Institute, Sichuan Cancer Center, School of Medicine, University of Electronic Science and Technology of China, Chengdu, China; ^4^ Department of Radiation Oncology, Beijing Shijitan Hospital, Capital Medical University, Ninth School of Clinical Medicine, Peking University, School of Oncology, Capital Medical University, Beijing, China; ^5^ Department of Thoracic Surgery, National Cancer Center/National Clinical Research Center for Cancer/Cancer Hospital, Chinese Academy of Medical Sciences and Peking Union Medical College, Beijing, China

**Keywords:** esophageal cancer, AJCC staging, prognostic model, neoadjuvant radiotherapy, neoadjuvant chemoradiotherapy

## Abstract

**Background:**

It is still uncertain whether the newly released eighth American Joint Committee on Cancer (AJCC) post-neoadjuvant pathologic (yp) tumor-node-metastasis (TNM) stage for esophageal carcinoma can perform well regarding patient stratification. The current study aimed to assess the prognostication ability of the eighth AJCC ypTNM staging system and attempted to explore how to facilitate the staging system for more effective evaluation of prognosis.

**Materials and methods:**

A total of 486 patients treated with neoadjuvant radiotherapy/chemoradiotherapy (nRT/CRT) were enrolled. ypN stage was reclassified by recursive partitioning. Prognostic performance, monotonicity, homogeneity, and discriminatory of yp and modified yp (myp) staging systems were assessed by time-dependent receiver operating characteristic (ROC), linear trend log-rank test, likelihood ratio χ2 test, Harrell’s c statistic, and Akaike information criterion (AIC).

**Results:**

The ypT stage, ypN stage, and pathologic response were significant prognostic factors of overall survival. Survival was not discriminated well using the eighth AJCC ypN stage and ypTNM stage. Recursive partitioning reclassified mypN0-N2 as metastasis in 0, 1–2, and ≥3 regional lymph nodes. Applying the ypT stage, mypN stage, and pathologic response to construct the myp staging system, the myp stage performed better in time-dependent ROC, linear trend log-rank test, likelihood ratio χ2 test, Harrell’s c statistic, and AIC.

**Conclusions:**

The eighth AJCC ypTNM staging system performed well in differentiating prognosis to some extent. By reclassifying the ypN stage and enrolling pathologic response as a staging element, the myp staging system holds significant potential for prognostic discrimination.

## Introduction

Esophageal carcinoma (EC) is associated with dismal prognosis and high rate of recurrence and, therefore, is among the most common causes of cancer-related mortality. For almost the past three decades, EC is the seventh most common cancer and the sixth cause of cancer-related deaths worldwide (1). Esophageal squamous cell carcinoma (ESCC) is the dominant histological subtype of EC (2); approximately 53% of all ESCC cases occur in China.

In the past few decades, as the level I evidence provided by the Chemoradiotherapy for Oesophageal Cancer Followed by Surgery Study (CROSS) (3) trial consolidated the role of neoadjuvant chemoradiotherapy (nCRT), multimodality therapy has been a common curative treatment approach in EC. The eighth edition of the American Joint Committee on Cancer (AJCC) tumor-node-metastasis (TNM) staging system for cancer of the esophagus and esophagogastric junction proposed the post-neoadjuvant pathologic (yp) stage for the first time (4–6). It was still unclear whether the newly released eighth AJCC yp staging system can perform well with respect to patient stratification.

Accurate evaluation of the staging system is essential for assessing prognosis and guiding stage-specific therapeutic strategy. The current study aimed to assess the prognostication ability of the eighth AJCC yp staging system as a prognostic tool in patients with ESCC undergoing radiotherapy/chemoradiotherapy (nRT/CRT) followed by esophagectomy and attempted to propose a refinement to facilitate the yp staging system for more effective prognostic evaluation.

## Materials and methods

### Patient population

This study was performed at the Cancer Hospital, Chinese Academy of Medical Science (CHCAMS). Between January 2007 and December 2017, a total of 486 patients with histopathologically confirmed ESCC treated with nRT/CRT followed by esophagectomy were enrolled. Patients were followed up to July 2019 or until death. The median follow-up duration was 62.2 months. All patients were staged according to the eighth AJCC yp staging system, and all the resection specimens have undergone pathologic review by the pathologists in our cancer center. Patient, tumor, and treatment characteristics were collected.

### Treatment

#### nRT/CRT

All patients received CT simulation, and their radiotherapy plans were developed and evaluated on the basis of CT images. The majority of patients received intensity-modulated radiation therapy (IMRT) (449/486, 92.4%), whereas the other patients received volumetric modulated arc therapy (37 of 486, 7.6%). Most patients (404 of 486, 83.1%) received conventionally fractionated radiotherapy, with a single dose of 1.8–2.0 Gy per fraction, a total of 36–50 Gy (median 40 Gy) to primary tumor, metastatic lymph nodes, and the involved lymphatic drainage region. A small number of patients (82 of 486, 16.9%) received simultaneous integrated boost–IMRT, with the boost dose of 2.10–2.21 Gy (median 2.14 Gy) per fraction, a total of 48.30–50 Gy (median 49.22Gy) to primary tumor and metastatic lymph nodes and the conventionally fractionated dose of 1.8–2.0 Gy (median 1.8 Gy) per fraction, a total of 41.4–46.0 Gy (median 41.4Gy) to the involved lymphatic drainage region. For those who are evaluated with potentially resectable lesions before receiving neoadjuvant treatment, a multidisciplinary team evaluation would be performed when the radiation dose achieved 40 Gy. Once evaluated as down-staging and converting to be with resectable lesions, surgical resection would be performed at 5–7 weeks after finishing neoadjuvant therapy. Patients enrolled in the current study were routinely evaluated by senior physicians in thoracic surgery, radiation oncology, and medical oncology before receiving neoadjuvant therapy. For those with lower tumor burden, initially evaluated with resectable lesions (146 of 486, 30.0% of all patients) and with favorable general status, concurrent CRT is considered preferable. Because of the retrospective nature of current study, the survival benefit of neoadjuvant chemoradiotherapy was being debated until the publication of the phase III CROSS trial (3). During the time period of 2007–2012, nRT is also considered as one of the alternative treatment approaches with acceptable toxicities and relative favorable radical resection rate, pathological response rate, and overall survival (OS) (7), especially to those who tended more likely to discontinue the concurrent chemoradiotherapy due to tumor status such as initially being considered with potentially resectable large, widespread lesions that needed neoadjuvant therapy to convert to be a resectable lesion (e.g., long primary tumor or multistation-regional lymph nodes metastases; 340 of 486, 70.0% of all patients), large planning target volume with accompanied relative high lung irritation volume, general status such as advanced age and presence of complications, or individual indication such as concerns about the treatment-related toxicities and preference for relative moderate treatment modality. Finally, 149 (30.7%) patients were treated with nCRT, comprising 122 platinum-paclitaxel cases, 18 5-fluorouracil-platinum cases, and nine other cases.

#### Surgical procedure

Sweet esophagectomy (262 cases, 53.9%), Ivor–Lewis esophagectomy (15 cases, 3.1%), McKeown esophagectomy (193 cases, 39.7%), and pharyngo-laryngo-esophagectomy (16 cases, 3.3%) were performed. All patients received standard abdominal lymphadenectomy (left and right paracardial regions, along the lesser curve and left gastric artery) and mediastinal lymphadenectomy (subcarinal, left and right bronchial, lower posterior mediastinum, pulmonary ligament, and paraesophageal). For patients who underwent right thoracotomy, paratracheal and left and right recurrent laryngeal nerve lymphadenectomy was performed. Cervical lymphadenectomy was systematically performed in the McKeown procedure. Patients with cervical EC were assigned to undergo pharyngo-laryngo-esophagectomy.

### Definition of the eighth ypTNM staging system

According to the eighth ypTNM staging system (5), the ypT stage was defined as follows: ypT0, no evidence of primary tumor; ypT1, tumor invades the lamina propria, muscularis mucosae, or submucosa; ypT2, tumor invades the muscularis propria; ypT3, tumor invades the adventitia; ypT4a, tumor invades the pleura, pericardium, azygos vein, diaphragm, or peritoneum; and ypT4b, tumor invades other adjacent structures, such as the aorta, vertebral body, or trachea. The ypN stage was defined as follows: ypN0, no regional lymph node metastasis; ypN1, metastasis in one to two regional lymph nodes; ypN2, metastasis in three to six regional lymph nodes; and ypN3, metastasis in ≥7 regional lymph nodes. The ypTNM stage was defined as follows: stage I: ypT0-2N0; stage II: ypT3N0; stage IIIA: ypT0-2N1; stage IIIB: ypT0-3N2, ypT3N1, and ypT4aN0; and stage IVA: ypT4aN1-2, ypT4b with any ypN status, and ypN3 with any ypT status.

### Assessment of pathologic response

According to the protocol for examination of specimens from patients with EC, the tumor regression score (TRG) (8) is recommended by the College of American Pathologists (CAP) for its concise description and good interobserver reproducibility among pathologists. The cancer regression grading system is defined as follows: TRG 0, no viable cancer cells; TRG 1, single cells or small groups; TRG 2, residual cancer with evidence of tumor regression, but more than single cells or rare small groups of cancer cells; and TRG 3, extensive residual cancer with no evidence of tumor regression. In the current study, the modified Ryan scheme for tumor regression score recommended by CAP was applied to assess response of tumor cells to nRT/CRT.

### Statistical analyses

OS time was calculated from the date of operation to the date of death or most recent follow-up. The Kaplan–Meier method was performed to estimate survival probabilities and the log-rank test for statistical comparisons in patient subgroups. Cox proportional hazards regression model was performed to investigate prognostic factors. The proportional hazards assumption was checked with the Schoenfeld’s global test before establishing the Cox regression model. All statistical tests were two-sided, and p < 0.05 was considered to indicate statistical significance.

Recursive partition analysis (RPA) can enroll both categorical and continuous variables, generate clinically more intuitive models that are easy to understand and derive the corresponding logical expression from the resulting decision tree, and perform relatively well in extrapolation. However, it is weak in dealing with missing data and may overfit data (9–11). In current study, on the basis of the pathological and survival information of the 486 patients in our cancer center, ypN stage groups were reclassified by RPA. Time-dependent receiver operating characteristic (ROC) was used to compare the prognostic performance of the staging systems. Monotonicity of staging systems was assessed using linear trend log-rank test. A larger χ2 value indicated greater efficacy in distinguishing between the ordered groups. The likelihood ratio χ2 test related to the Cox regression model was used to measure homogeneity; a higher ratio was indicative of more homogeneity in a group. Discriminatory ability was quantified using Harrell’s c statistic. A value of 0.5 refers to random prediction, and a value of 1 refers to perfect discrimination. The Akaike information criterion (AIC) of Cox proportional hazards model was used to minimize potential bias in comparing different prognostic systems, defined as follows: AIC = −2 log likelihood + 2 × (the number of parameters in a model). A smaller AIC value indicated that the model performed better in discrimination.

All statistical calculations were performed with R software, version 3.6.2 (R Foundation for Statistical Computing, Vienna, Austria).

## Results

### Patient characteristics

In the current study, 486 patients with pathologically confirmed ESCC treated with nRT/CRT followed by esophagectomy from 1980 through 2017 were enrolled. Male patients accounted for the majority (81.9% of all cases). Almost two-third patients were <60 years old. The Eastern Cooperative Oncology Group performance score of 90.9% and 9.1% of the patients was 0 and 1, respectively. In addition, 43%, 24.5%, 9.0%, 16.5%, and 7.0% of the patients were diagnosed with eighth AJCC yp stage I, II, IIIA, IIIB, and IVA, whereas 43.4%, 36.0%, and 20.6% of the lesions were classified as TRG 0–1, 2, and 3. All clinicopathologic characteristics are listed in **Table 1**. The 1-, 3-, and 5-year OS was 84.2%, 55.3%, and 45.6%, respectively, and the median survival time was 43.7 months.

**Table 1:** Baseline characteristics.

Characteristic		No. (%)		Characteristic		No. (%)
Age (years) (range 27-78 years)	<60 years	326 (67.1%)		Tumor length (cm) (range, 1-22 cm)	<5cm	188 (38.8%)
	≥60 years	160 (32.9%)			≥5cm	298 (61.2%)
	Median (IQR)	56 (11)			Median (IQR)	5.0 (2.0)
Sex	Male	398 (81.9%)		Tumor location	Proximal third	124 (25.5%)
	Female	88 (18.1%)			Middle third	307 (63.2%)
Pre-treatment 6th AJCC T stage	T1	10 (2.1%)			Distal third	55 (11.3%)
	T2	37 (7.6%)		8th AJCC ypT stage	T0	107 (22.0%)
	T3	210 (43.2%)			T1	46 (9.5%)
	T4	229 (47.1%)			T2	119 (24.5%)
Pre-treatment 6th AJCC N stage	N0	81 (16.7%)			T3	181 (37.2%)
	N1	405 (83.3%)			T4a	24 (4.9%)
Pre-treatment 6th AJCC M stage	M0	470 (96.7%)			T4b	9 (1.9%)
	M1a	7 (1.4%)		8th AJCC ypN stage	N0	342 (70.4%)
	M1b	9 (1.9%)			N1	95 (19.5%)
Pre-treatment 6th AJCC TNM stage	Stage IIA	45 (9.3%)			N2	32 (6.6%)
	Stage IIB	39 (8.0%)			N3	17 (3.5%)
	Stage III	386 (79.4%)		8th AJCC ypTNM stage	Stage I	208 (42.8%)
	Stage IVA	7 (1.4%)			Stage II	120 (24.7%)
	Stage IVB	9 (1.9%)			Stage IIIA	43 (8.8%)
Radiation dose (Gy) (range, 30-50 Gy)	≤40Gy	359 (73.9%)			Stage IIIB	80 (16.5%)
	>40Gy	127 (26.1%)			Stage IVA	35 (7.2%)
	Median (IQR)	40 (1.4)		Pathologic response	TRG 0-1	213 (43.8%)
Concurrent chemotherapy	No	337 (69.3%)			TRG 2	173 (35.6%)
	Yes	149 (30.7%)			TRG 3	100 (20.6%)
Surgical procedure	Sweet	262 (53.9%)		Carcinoma cell embolus	No	462 (95.1%)
	Ivor-Lewis	15 (3.1%)			Yes	24 (4.9%)
	McKeown	193 (39.7%)				
	PLE	16 (3.3%)				

PLE, Pharyngo-Laryngo-Esophagectomy.

### Prognostic factors

All clinicopathologic characteristics in **Table 1** were enrolled in log-rank univariable analysis. Univariable analysis showed that sex, radiation technique, radiation dose, administration of concurrent chemotherapy, surgical procedure, eighth AJCC ypT stage, ypN stage, ypTNM stage, and pathologic response were related to OS (**Supplementary Table 1**). Then, we enrolled the factors that were related to OS in the univariable analysis into the Cox multivariable analysis. Results of multivariable analysis are shown in the forest plots (**Supplementary Figure 1**). Administration of chemotherapy, the depth of primary lesion invasion (ypT stage), the number of metastatic regional lymph nodes (ypN stage), and the pathologic response were prognostic factors of OS.

For 149 patients receiving nCRT (**Supplementary Table 2**), the 5-year OS was not significantly different between patients receiving taxel-platinum (TP), platinum-fluorouracil (PF), and other regimes (P = 0.221); patients completing 1–3 weekly cycles and 4–5 weekly cycles (P = 0.100); and patients completing one and two 21-day cycles (P = 0.717). As for the treatment related toxicities, 9.5% (46 of 486) of the patients encountered anastomotic fistula. The other incidence of adverse events was listed in **Supplementary Table 3**. The 5-year OS was not significantly different between patients encountered grade 3 or higher adverse events or anastomotic fistula and those did not encountered grade 3 or higher adverse events and anastomotic fistula (42.1% vs. 46.5%, P = 0.204).

### Assessment of the eighth AJCC ypTNM staging system

Comparison of OS between the eighth AJCC ypT stage groups through the Kaplan–Meier cumulative survival probability curve and log-rank method (**Supplementary Figure 2** and **Supplementary Table 4**) showed significant differences among the four prognostic groups. Furthermore, the OS monotonically decreased with higher ypT stage.

**Figure 1:**
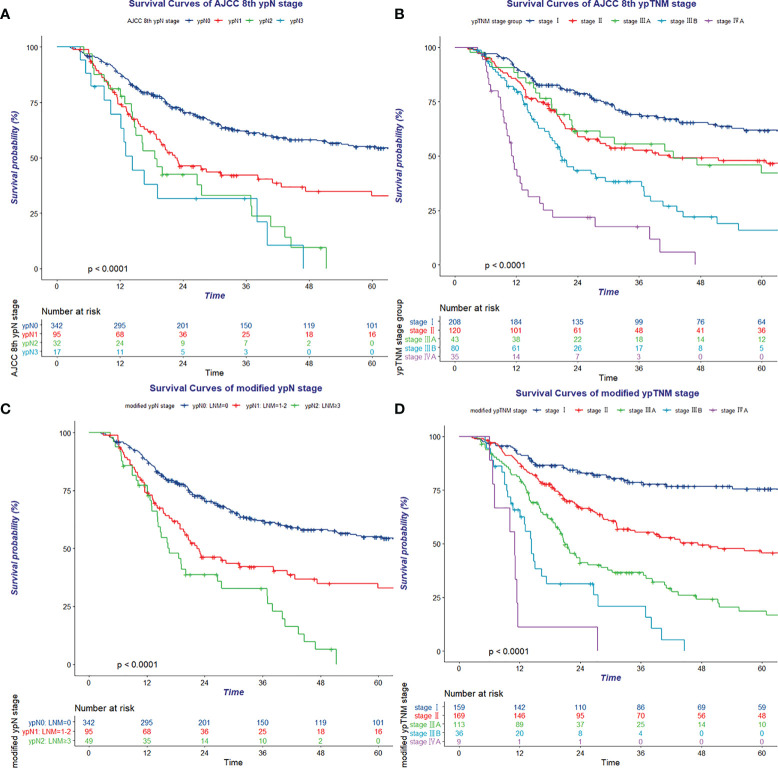
Kaplan–Meier survival curve of eighth AJCC ypN stage **(A)**, eighth AJCC ypTNM stage **(B)**, modified ypN stage **(C)**, and modified ypTNM stage **(D)**.

The Kaplan–Meier cumulative survival probability curve and log-rank test of the eighth AJCC ypN stage groups indicated that survival could not be well distinguished by the eighth AJCC ypN stage groups (ypN1 vs. ypN2, p = 0.059; and ypN2 vs. ypN3, p = 0.369; **Figure 1A** and **Supplementary Table 5**). The median number of resected lymph nodes and pathologic positive lymph nodes were 14 and 0, respectively. A total of 342 (70.4%), 65 (13.4%), and 30 (6.2%) patients were diagnosed with 0, 1, and 2 metastatic lymph nodes, respectively [total of 437 cases (90.0%)]. Furthermore, 17 (3.5%), 5 (1.0%), 7 (1.4%), 3 (0.6%), and 17 (3.5%) patients were diagnosed with 3, 4, 5, 6, and >7 metastatic lymph nodes, respectively (**Supplementary Table 6**).

Likewise, survival was not well discriminated between stage II and stage IIIA (p = 0.929) using the eighth ypTNM staging system in the Kaplan–Meier cumulative survival probability curve and log-rank test (**Figure 1B** and **Supplementary Table 7**).

### Refinement of the ypTNM staging system

**Figure 2:**
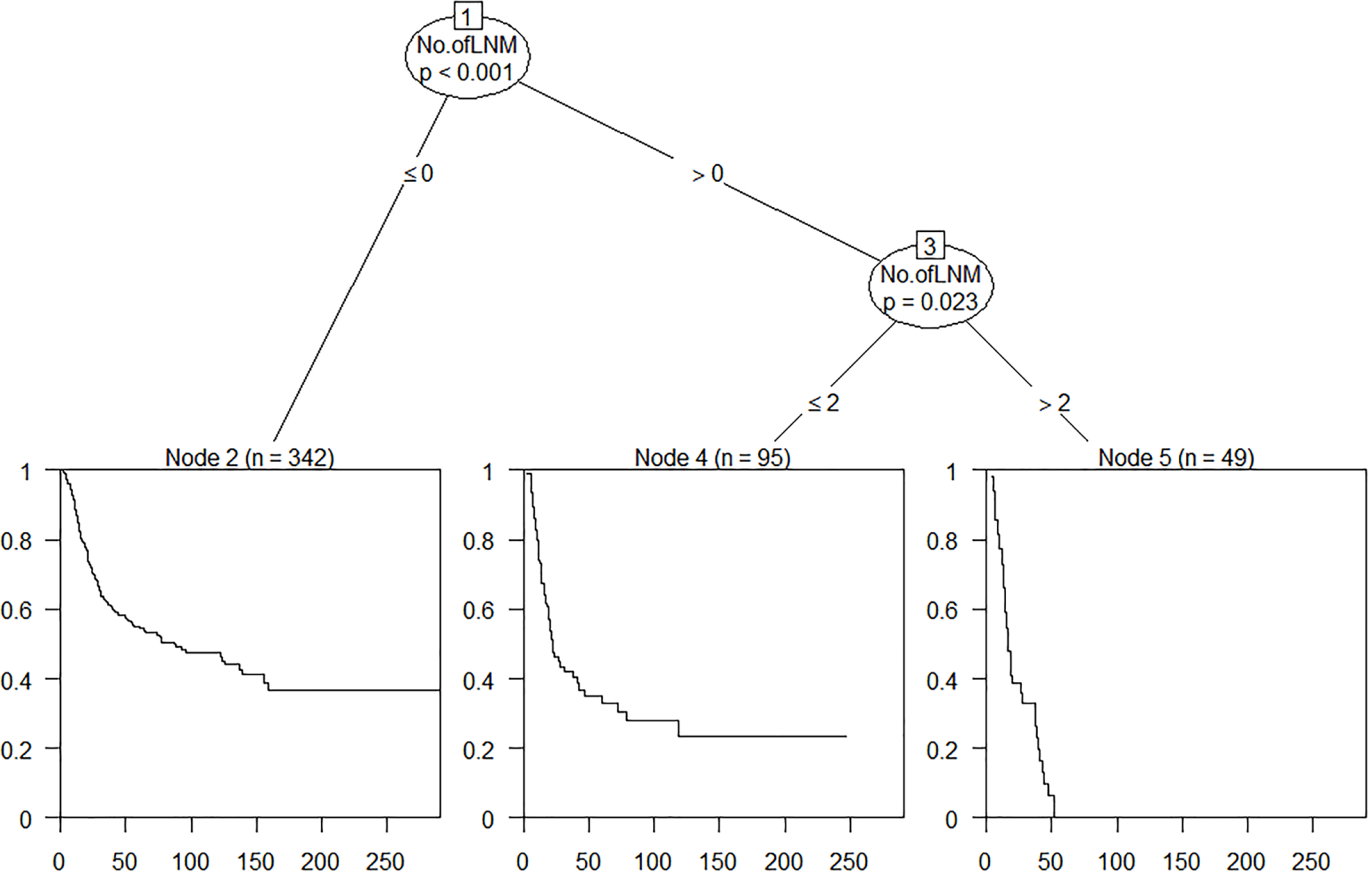
Classification tree of the number of metastatic lymph nodes.

On the basis of data of this observed cohort, OS time, OS status, and the number of pathologically confirmed positive lymph nodes were enrolled in RPA and used to define the best grouping for discriminating patients with different prognosis (**Figure 2**). All patients were divided into three prognostic groups and therefore hypothesized the modified ypN (mypN) stage as follows: mypN0, no regional lymph node metastasis; mypN1, metastasis in one to two regional lymph nodes; and mypN2, metastasis in ≥3 regional lymph nodes. The Kaplan–Meier cumulative survival probability curve and log-rank test showed that the survival between any two mypN staging groups was significantly different (**Figure 1C** and **Supplementary Table 8**).

In addition to the ypT and ypN stage, pathologic response was another significant prognostic factor revealed in multivariate analysis. OS was discriminated well between TRG 0–1, TRG 2, and TRG 3 groups (64.9% vs. 36.7% vs. 18.4%, p < 0.001). Subgroup analysis indicated that the pathologic response significantly influenced survival in the ypT0-2N0 (p = 0.012), ypT3N0 (p < 0.001), and ypT0-2N1 (p = 0.025) groups (**Supplement Figure 3**). Using 0.15 as the cutoff log rank p-value to combine prognosis subgroups with similar prognosis as a modified yp (myp) stage group (**Supplementary Table 9**). The myp stage was defined as follows: stage I: mypT0-2N0 TRG 0–1 and mypT3N0 TRG 0–1; stage II: mypT0-2N0 TRG 2–3, mypT3N0 TRG 2, and mypT0-2N1 TRG 0–2; stage IIIA: mypT3N0 TRG 3, mypT0-2N1 TRG 3, mypT0-2N2, mypT3N1, and mypT4aN0; stage IIIB: mypT3N2 and mypT4aN1-2; and stage IVA: ypT4b with any mypN status (**Supplementary Figure 4**). The Kaplan–Meier cumulative survival probability curve and log-rank test showed that all the myp stage groups were well discriminated from each other (**Figure 1D** and **Supplementary Table 10**). In addition, for patients treated with nRT/nCRT, the Kaplan–Meier cumulative survival probability curve and log-rank test showed that ypT stage, mypN stage, TRG, and mypTNM stage could perform well in discrimination (**Supplementary Figure 5**).

**Figure 3:**
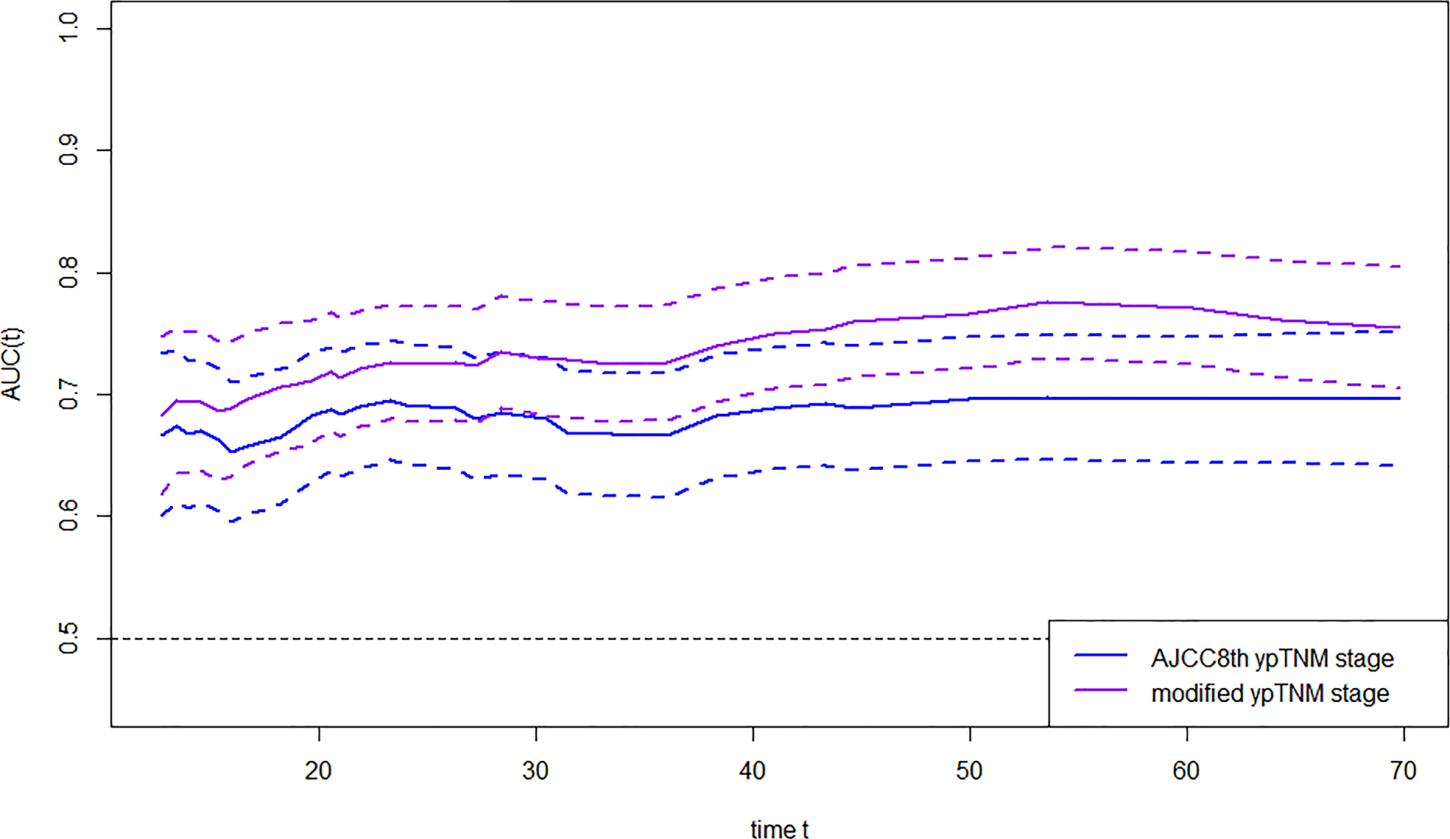
AUC of AJCC eighth and modified ypTNM stage in time-dependent ROC.

The OS time, OS status, and eighth AJCC yp stage/myp stage were used to construct survival regression, and time-dependent ROC analysis was employed to compare AUC between the two staging systems at certain times (**Figure 3**). AUC of the myp stage was significantly higher than that of the eighth AJCC ypTNM stage since the third year after receipt of treatment [72.63 vs. 66.60 at the third year (p = 0.002), 76.49 vs. 69.75 at the fourth year (p = 0.002), and 77.06 vs. 69.59 at the fifth year (p = 0.001)] (**Supplementary Table 11**). In terms of monotonicity, the linear trend log-rank χ2 was higher in the myp stage than that in the eighth AJCC ypTNM stage (127.5 vs. 80.3). As for homogeneity, the likelihood ratio χ2 test related to the Cox regression model was higher in the myp stage than that in the eighth AJCC ypTNM stage (113.2 vs. 76.4). Meanwhile, Harrell’s c statistic was larger (0.68 vs. 0.64), and AIC was smaller (2,688.56 vs. 2,725.39) in the myp stage (**Table 2**), both revealed better performance in discrimination.

**Table 2:** Comparison of eighth AJCC TNM stage and modified ypTNM stage.

ypTNM stage	Monotonicity	Homogeneity	Discriminatory	
	Linear trend log-rank χ^2^	Likelihood ratio χ^2^	AIC	Harrell’s C statistic
Eighth AJCC ypTNM stage	80.3	76.4	2,725.39	0.64
Modified ypTNM stage	127.5	113.2	2,688.56	0.68

AIC, Akaike information criterion.

## Discussion

The prognosis of patients who treated with neoadjuvant therapy was different from those who treated with upfront surgical resection without neoadjuvant therapy but pathologically diagnosed with similar stage (12). It was necessary to group the patients receiving neoadjuvant therapy and those receiving upfront surgical resection into different staging system. The eighth AJCC TNM staging system for cancer of the esophagus and esophagogastric junction proposed the yp stage for the first time and made considerable progress in improving the prognostic ability of the staging system. Meanwhile, the ypT stage and ypN stage could be obtained at early time and therefore enable early prognostic evaluation and prompt clinical application of potential treatments for patients. However, according to the recommendation of the Worldwide Esophageal Cancer Collaboration (WECC) (4), the yp stage groups comprised the ypT stage groups (ypT0-2, ypT3, ypT4a, and ypT4b) different from the seventh and eighth AJCC pathologic T stage groups and ypN stage groups (ypN0, ypN1, ypN2, and ypN3) consistent with the seventh and eighth AJCC pathologic N stage groups. The analysis results of the WECC yp stages indicated that survival was not well discriminated between ypN2 and ypN3 in the ESCC subgroup (6), which might therefore lower the prognostication ability of the yp staging system, given that squamous cell carcinoma is the histologically dominant subtype of EC and had great influence on the staging system. Moreover, the prognostication ability of yp staging system might be limited because it enrolled anatomic factors (ypT stage and ypN stage) only, without other prognostic factors. Prognosis of patients with EC depends on the complex interplay of TNM classifications and non-anatomic factors (13). Several previous studies (14–16) have revealed that prognosis of patients who received nCRT followed by esophagectomy was varied from pathologic responders to non-responders. The survival of pathologic non-responders was equivalent to or even worse than patients who received primary esophagectomy. By contrast, pathologic responders attained better prognosis. Therefore, adopting the level of pathologic response to be a staging factor might be a feasible scheme to improve the prognostication ability of the TNM staging system. Whether the new staging system can perform well with respect to patient stratification is still uncertain. Validation against external data to those used for developing the system is important. The present study aimed to serve as an external validation of the newly released staging system for patient prognosis and to provide evidence to facilitate the subsequent ninth AJCC staging system for more effective stratification of patients with various outcomes.

In this study, the Kaplan–Meier cumulative survival probability curve and log-rank test showed that prognosis between any two eighth AJCC ypT stage groups (ypT0-2, ypT3, ypT4a, and ypT4b) was significantly different, in accordance with a previous study by Wang et al. (17). The eighth AJCC ypT stage groups performed well with respect to identifying patients with different prognosis. However, there was no significant difference in OS between the ypN1 and ypN2 groups as well as between the ypN2 and ypN3 groups through our validation, in concordance with results of previous studies by Wang et al. (18), Shao et al. (19), and Sisic et al. (20). Survival was not significantly different between ypN groups, which is consistent with the seventh and eighth AJCC pathologic N stage groups. Likewise, the results of WECC yp stage analysis indicated that survival was not well discriminated between ypN2 and ypN3 in the ESCC subgroup (6). Hence, directly adopting the seventh and eighth pathologic N grouping system might not be the best solution to distinguish patients with different prognosis. Several previous studies (6, 21–23) have shown that both the number of lymph nodes harvested in surgery and the number of diagnosed as metastatic lesions in pathologic assessment after neoadjuvant therapy followed by surgery were lesser than those in surgery alone. Data from WECC demonstrated that the proportion of patients with zero, one, and two metastatic lesions after neoadjuvant therapy was 70%, 13%, and 6.4%, respectively, in the ESCC group. Analogously, the majority of patients were diagnosed with zero [342 cases (70.4%)], one [65 cases (13.4%)], and two (30 cases (6.2%)] metastatic lymph nodes in the current study. The phenomenon of the number of metastatic lymph nodes after neoadjuvant therapy tended to be lesser than those in surgery alone, and the nature of SCC tended to be with less regional metastatic lymph nodes and more visible pathologic response (21, 24) might be responsible for lack of discrimination when applying the eighth AJCC ypN stage groups that are completely identical with pN stage groups. In the current study, RPA-based mypN prognostic groups showed better discrimination than the eighth AJCC ypN stage groups, as survival was significantly different between any two groups in the log-rank test. Reclassifying ypN stage considering OS time, OS status, and the number of metastatic lesion facilitated the prognostication ability of the staging system.

Several previous studies aimed to evaluate the discriminatory ability of the eighth AJCC yp staging system showed that survival was less distinctive between several ypTNM groups (20, 25, 26), consistent with the results of our study. Proposing an exclusive yp staging system according to ypT and ypN status seems inadequate. Adopting other survival-affected nonanatomic tumor characteristics as staging factors might be a feasible scheme to improve the prognostication ability of the staging system. Swisher et al. proposed that the extent of pathologic response following CRT was an independent risk factor for survival and should be incorporated in the pTNM staging system to better predict patient outcome in EC (27). Francis et al. studied the association between histopathologic tumor viability (HTV) on long-term survival and recurrence rates for esophageal adenocarcinoma patients treated with neoadjuvant therapy and noted that tumor viability may either prove a strong enough prognostic indicator to be an adjunct to ypT-descriptor or perhaps replace tumor depth altogether in a revised ypTNM staging system; they suggested that HTV may be a practical early endpoint predicting treatment efficacy (28). Xi et al. revealed that pathologic complete response and TNM stage were the independent prognostic factors of esophageal adenocarcinoma and proposed a recurrence risk stratification system based on pathologic response and TNM stage for risk-based postoperative surveillance strategies (29). In the current study, Cox proportional hazards regression model–based multivariate analysis revealed that pathologic response significantly influenced survival in addition to the ypT stage and ypN stage. Enrolling ypT stage, reclassified mypN stage, and modified Ryan scheme for tumor regression score to establish myp stages, time-dependent ROC analysis revealed that the myp stages performed better in prognostication. Adding pathologic response as an element into ypTNM staging seemed a feasible scheme facilitating the yp staging system as a more effective prognostic tool.

The ypTNM staging system is deemed to be a prognostic model that could distinguish patients with different prognosis at early time. In the current study, we reclarified the ypN stage groups and added the non-anatomic factor, TRG, to the ypTNM staging system to facilitate its prognostic ability. Because the addition of TRG could further improve the predictive performance of ypTNM stage and it could be obtained from the pathological examination after surgical resection, the modified classification system in the current study could be attained early, processed strong correlation with OS, and could be meaningful to assess prognosis in the early phase and to administer individual therapeutic strategies accordingly. Several previous studies demonstrated that, except for the number of positive lymph nodes, the positive lymph node ratio and the number of positive lymph node stations could also perform well in prognosis evaluation (30–32). It is essential to enroll these metastatic lymph nodes information to establish a mypN stage and further improve the prognostic ability of the modified classification system. Meanwhile, other non-anatomic factors, which could be obtained from pathological specimens or peripheral blood at early time, might also be the potential modified staging system elements.

The main limitation of this study was its retrospective nature, which may have introduced some bias in the results and conclusion. Thus, the results should be validated in another prospective data set. Moreover, because cases were collected over a relatively large time span, uncertain confounding factors still existed, partially owing to the difference in clinical staging modalities and therapeutic strategies. Moreover, a consensus on the standard in evaluating the pathologic response of patients with EC who received neoadjuvant therapy has to be reached. Once an appropriate, applicable, and reproducible pathologic response evaluation standard is proposed, its inclusion in staging nomenclature can be considered.

The survival of patients receiving nRT/CRT followed by esophagectomy is strongly influenced by the ypT stages, ypN stages, and pathologic response. It is reasonable to propose the yp staging system according to the ypT status, ypN status, and pathologic response as another staging element to facilitate the prognostication ability of the yp staging system.

## Data availability statement

The raw data supporting the conclusions of this article will be made available by the authors, without undue reservation.

## Ethics statement

Written informed consent was obtained from the individual(s) for the publication of any potentially identifiable images or data included in this article.

## Author contributions

WH: Methodology, validation, formal analysis, investigation, data curation, and writing—original draft. ZZ, JuL, DC, QF, NB, TZ, XW, LD, WW, WL, JW, QX, YM, XL, DF, JiL, DW, and JZ: Resources and investigation. WD, QW, WN, and CL: Investigation and data curation. ZX: Conceptualization, methodology, validation, and writing—review and editing. All authors contributed to the article and approved the submitted version.

## Funding

This study was supported by the Beijing Hope Run Special Fund of the Cancer Foundation of China (LC2016L04). The funder of the study had no role in study design, data collection, data analysis, data interpretation, or writing of the report.

## Conflict of interest

The authors declare that the research was conducted in the absence of any commercial or financial relationships that could be construed as a potential conflict of interest.

## Publisher’s note

All claims expressed in this article are solely those of the authors and do not necessarily represent those of their affiliated organizations, or those of the publisher, the editors and the reviewers. Any product that may be evaluated in this article, or claim that may be made by its manufacturer, is not guaranteed or endorsed by the publisher.
