# Supplementary material for: Applying post-neoadjuvant pathologic stage as prognostic tool in esophageal squamous cell carcinoma
Source: Front Oncol. 2022 Nov 10;12:998238. doi: 10.3389/fonc.2022.998238 (PMC9685303; doi:10.3389/fonc.2022.998238)
Supplement: Supplementary file 1 [file DataSheet_1.pdf]

Supplementary Materials

Supplementary Tables

Supplementary table 1. log-rank univariable analysis

Characteristic	$\chi 2$	P value
Age (<60 years vs. ≥60 years)	0.007	0.934
Sex (Male vs. Female)	4.856	0.028
ECOG performance score (0 vs. 1)	1.810	0.179
Pre-treatment 6th AJCC T stage (T1 vs. T2 vs. T3 vs. T4)	2.025	0.567
Pre-treatment 6th AJCC N stage (N0 vs. N1)	0.637	0.425
Pre-treatment 6th AJCC M stage (M0 vs. M1a vs. M1b)	5.259	0.072
Pre-treatment 6th AJCC TNM stage (stage IIA vs. stage IIB vs. stage III vs. stage IVA vs. stage IVB)	5.631	0.228
Radiation dose (≤40Gy vs. >40Gy)	5.095	0.024
Concurrent chemotherapy (No vs. Yes)	17.312	<0.001
Surgical procedure (Sweet vs. Ivor-Lewis vs. McKeown vs. PLE)	11.850	0.008
Tumor length (<5cm vs. ≥5cm)	0.515	0.473
Tumor location (Proximal third vs. Middle third vs. Distal third)	0.687	0.709
8th AJCC ypT stage (T0 vs. T1 vs. T2 vs. T3 vs. T4a vs. T4b)	74.360	<0.001
8th AJCC ypN stage (N0 vs. N1 vs. N2 vs. N3)	53.646	<0.001
8th AJCC ypTNM stage (stage I vs. stage II vs. stage IIIA vs. stage IIIB vs. stage IVA)	103.059	<0.001
Pathologic response (TRG 1 vs. TRG 2 vs. TRG 3)	56.788	<0.001
Carcinoma cell embolus (No vs. Yes)	1.834	0.176

* ECOG=Eastern Cooperative Oncology Group, AJCC=American Joint Committee on Cancer, PLE=Pharyngo-Laryngo-Esophagectomy.

Supplementary table 2. Chemotherapy-related characteristics

Characteristic		No. (%)	5-year OS	Log-rank P value
Chemotherapy regimes				
TP		121(81.2%)	62.8%	0.221
PF		19(12.8%)	61.4%	
Other		9(6.0%)	64.8%	
Chemotherapy completion				
Weekly cycle modality	1-3 weeks	46(39.0%)	48.7%	0.100
	4-5 weeks	72(61.0%)	65.2%	
21-days cycle modality	1 cycle	16(51.6%)	52.1%	0.717
	2 cycles	15(48.4%)	57.8%	

* TP= platinum-paclitaxel, PF=5-fluorouracil- platinum.

Supplementary table 3. Incidence of adverse events

Adverse events	Grade, No. (%)				
	1	2	3	4	5
Anemia	36(7.4%)	6(1.2%)	0(0.0%)	0(0.0%)	0(0.0%)
Leukopenia	154(31.7%)	169(34.8%)	34(7.0%)	2(0.4%)	0(0.0%)
Thrombocytopenia	55(11.3%)	17(3.5%)	7(1.4%)	2(0.4%)	0(0.0%)
Hepatic insufficiency	2(0.4%)	1(0.2%)	1(0.2%)	0(0.0%)	0(0.0%)
Nausea	64(13.2%)	34(7.0%)	6(1.2%)	0(0.0%)	0(0.0%)
Vomiting	20(4.1%)	10(2.1%)	0(0.0%)	0(0.0%)	0(0.0%)
Esophagitis	345(71.0%)	117(24.1%)	8(1.6%)	0(0.0%)	0(0.0%)
Pneumonitis	4(0.8%)	2(0.4%)	1(0.2%)	1(0.2%)	0(0.0%)
Fever	16(3.3%)	10(2.1%)	4(0.8%)	0(0.0%)	0(0.0%)

Supplementary table 4. log-rank test of AJCC 8th ypT stage groups

ypT stage	No. (%)	MST (months)	5-year OS	χ^2	P value
T0-2	272(56.0%)	not reach	53.9%		
T3	181(37.2%)	28.156	39.7%	16.102*	<0.001*
T4a	24(4.9%)	20.698	8.4%	4.771*	0.029*
T4b	9(1.9%)	11.006	0%	4.763*	0.029*
Overall	486(100%)	43.729	45.6%	69.938	<0.001

*log-rank test with the ypT stage group above.

Supplementary table 5. log-rank test of AJCC 8th ypN stage

ypN stage	No. (%)	MST (months)	5-year OS	χ^2	P value
N0	342(70.4%)	not reach	55.1%		
N1	95(19.5%)	22.439	32.8%	17.266*	<0.001*
N2	32(6.6%)	18.595	0%	3.572*	0.059*
N3	17(3.5%)	14.324	0%	0.805*	0.369*
Overall	486(100%)	43.729	45.6%	53.646	<0.001

*log-rank test with the ypN stage above.

Supplementary table 6. distribution of the pathologic positive lymph nodes No. groups in the study population

No. of pathologic positive lymph nodes	Cases (%)
0	342(70.4%)
1	65(13.4%)
2	30(6.2%)
3	17(3.5%)
4	5(1.0%)
5	7(1.4%)
6	3(0.6%)
≥7	17(3.5%)

Supplementary table 7. log-rank test of AJCC 8th ypTNM stage

ypTN stage	No.(%)	MST(months)	3-year OS	5-year OS	χ^2	P value
Stage I	208(42.8%)	not reach	69.2%	61.8%		
Stage II	120(24.7%)	42.119	52.6%	48.0%	8.955*	0.003*
Stage IIIA	43(8.8%)	42.710	55.6%	42.3%	0.008*	0.929*
Stage IIIB	80(16.5%)	20.698	38.3%	15.8%	6.077*	0.014*
Stage IVA	35(7.2%)	11.466	17.5%	0%	12.728*	＜0.001*
Overall	486(100%)	43.729	55.3%	45.6%	103.059	＜0.001

*log-rank test with the ypTNM stage above.

Supplementary table 8. log-rank test of modified ypN stage

ypN stage	No.(%)	MST(months)	5-year OS	χ^2	P value
N0	343(70.4%)	not reach	55.1%		
N1	95(19.5%)	22.439	32.8%	17.266*	＜0.001*
N2	49(10.1%)	16.624	0%	6.850*	0.009*
Overall	487(100%)	43.729	45.6%	52.631	＜0.001

*log-rank test with the modified ypN stage above.

Supplementary table 9. log-rank test of modified ypTN stage groups

ypTN stage	No.(%)	MST(months)	3-year OS	5-year OS	χ^2	P value
ypT0-2N0 TRG1	136(28.0%)	not reach	77.0%	73.0%		
ypT3N0 TRG1	24(4.9%)	not reach	87.3%	87.3%	1.075*	0.300*
ypT0-2N1 TRG1	25(5.1%)	not reach	62.6%	57.3%	2.907*	0.088*
ypT0-2N0 TRG2	57(11.7%)	49.544	58.7%	47.5%	0.048*	0.826*
ypT0-2N0 TRG3	15(3.1%)	26.875	35.7%	23.8%	0.877*	0.349*
ypT3N0 TRG2	62(12.8%)	38.604	51.3%	46.5%	0.080*	0.777*
ypT0-2N1 TRG2	9(1.9%)	42.710	62.2%	31.1%	0.018*	0.893*
ypT3N0 TRG3	34(7.0%)	21.585	29.7%	21.2%	2.150*	0.143*
ypT0-2N1 TRG3	9(1.9%)	19.910	26.7%	0%	0.544*	0.461*
ypT3N1	37(7.6%)	20.632	39.5%	33.8%	0.537*	0.464*
ypT4aN0	12(2.5%)	29.405	43.2%	14.4%	0.020*	0.888*
ypT0-2N2	21(4.3%)	19.121	42.9%	0%	0.920*	0.338*
ypT4aN1-2	12(2.5%)	9.593	24.7%	0%	4.108*	0.043*
ypT3N2	24(4.9%)	14.324	20.9%	0%	1.258*	0.262*
ypT4b anyN	9(1.8%)	11.006	0%	0%	7.937*	0.005*
Overall	486(100%)	43.729	55.3%	45.6%	155.187	＜0.001

*log-rank test with the prognostic group above.

Supplementary table 10. log-rank test of modified ypTNM stage

ypTN stage	No.(%)	MST(months)	3-year OS	5-year OS	χ^2	P value
Stage I	159(32.7%)	not reach	78.6%	75.5%		
Stage II	169(34.8%)	47.343	55.3%	45.8%	16.887*	＜0.001*
Stage IIIA	113(23.3%)	20.994	36.6%	18.6%	21.560*	＜0.001*
Stage IIIB	36(7.4%)	14.292	20.9%	0%	8.768*	0.003*
Stage IVA	9(1.9%)	11.006	0%	0%	5.287*	0.021*
Overall	486(100%)	43.729	55.3%	45.6%	147.343	＜0.001

*log-rank test with the modified ypTNM stage above.

Supplementary table 11. AUC of 8th AJCC ypTNM stage and modified ypTNM stage in time dependent ROC

ypTNM stage	AUC,%,1 year (95%CI)	AUC,%,2 years (95%CI)	AUC,%,3 years (95%CI)	AUC,%,4 years (95%CI)	AUC,%,5 years (95%CI)
AJCC 8th ypTNM stage	66.84 (59.96,73.73)	69.34 (64.43,74.25)	66.60 (61.48,71.71)	69.75 (64.69,74.80)	69.59 (64.40,74.78)
Modified ypTNM stage	68.03 (61.31,74.75)	72.57 (67.89,77.24)	72.63 (67.89,77.36)	76.49 (71.96,81.03)	77.06 (72.44,81.68)
Adjusted P value	0.924	0.124	0.002	0.002	0.001

*AUC=area under curve, ROC=receiver operating characteristic.

Supplementary Figures

Supplementary figure 1 Forestplot of Cox proportional hazard regression multivariable analysis.

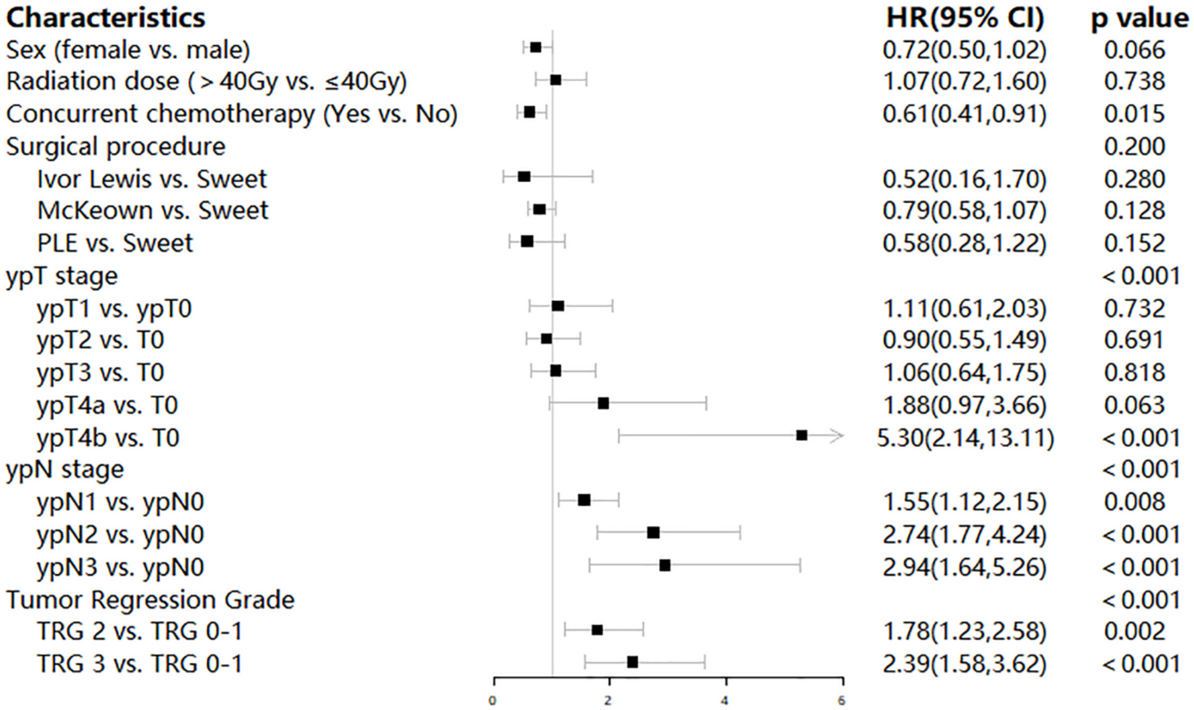

Supplementary figure 2 Kaplan-Meier survival curve of 8th AJCC ypT stage groups.

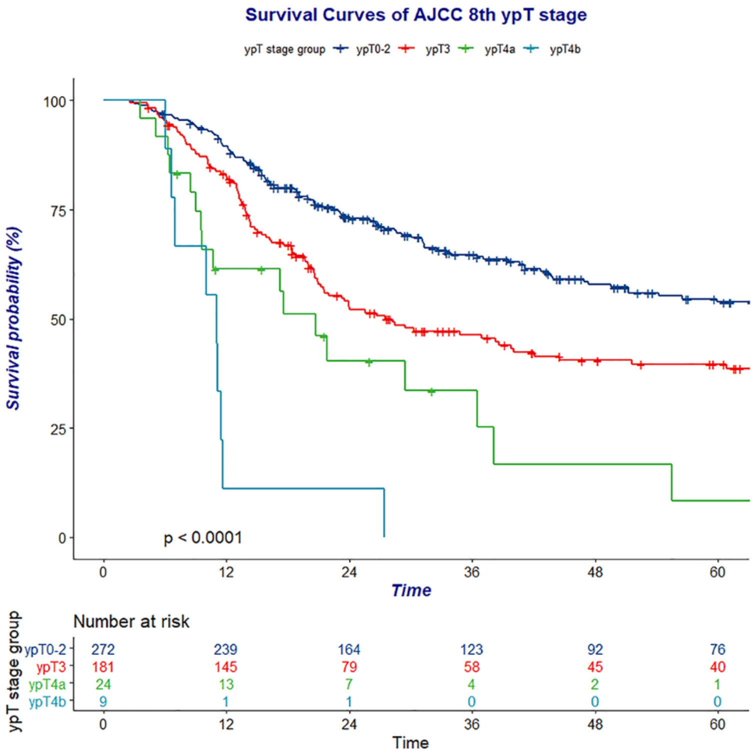

Supplementary figure 3 Kaplan-Meier survival curve of pathologic response in ypTN stage groups A) ypT0-2N0, B) ypT3N0,C) ypT0-2N1, D) ypT3N1, E) ypT4aN0, F) ypT0-2N2, G) ypT4aN1-2, H) ypT3N2, I) ypT4b anyN.

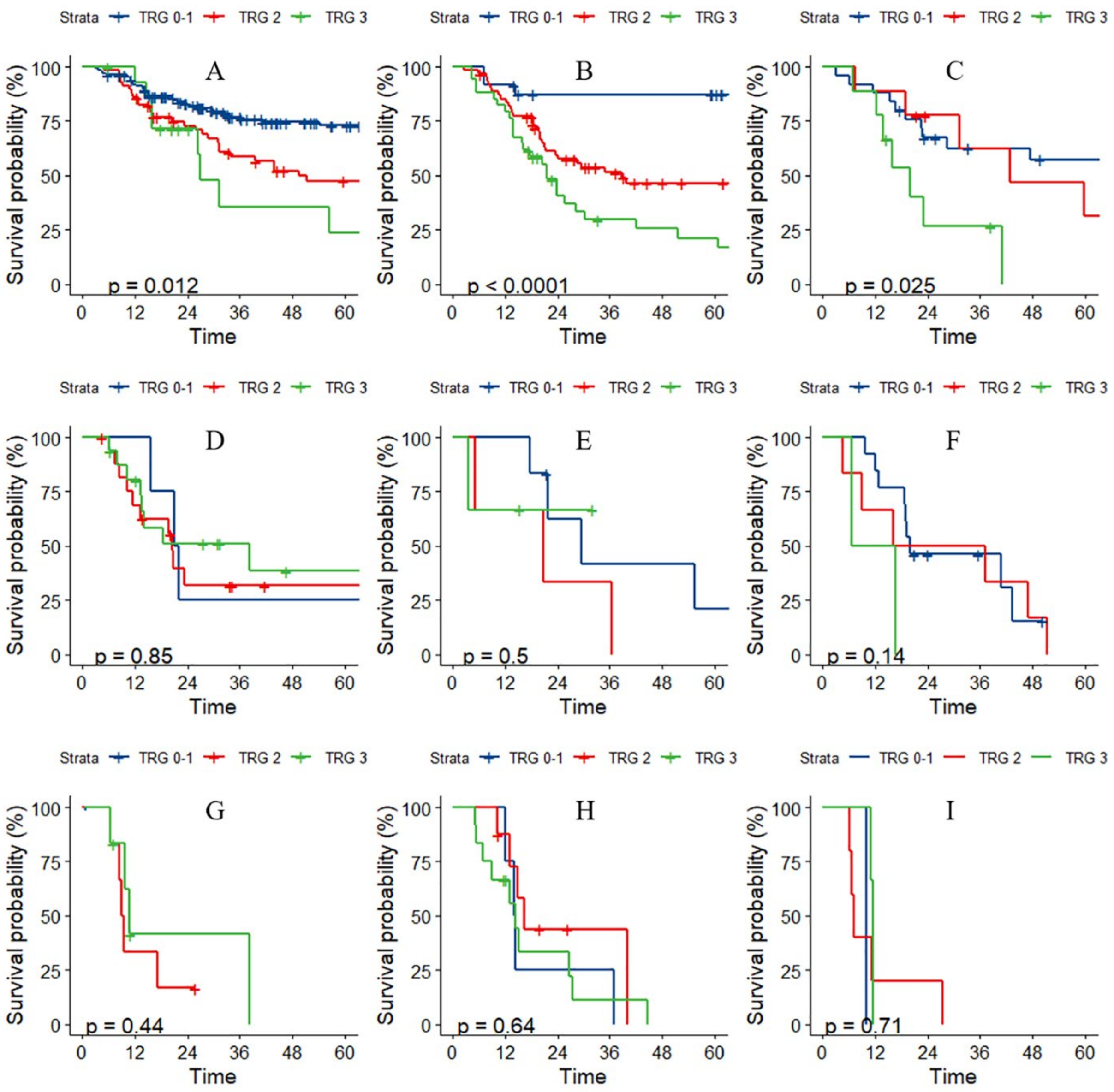

Supplementary figure 4 The 8th AJCC ypTNM stage (A) and modified yp stage (B).

	N0	N1	N2	N3
T0-2	I	IIIA	IIIB	IVA
T3	II	IIIB	IIIB	IVA
T4a	IIIB	IVA	IVA	IVA
T4b	IVA	IVA	IVA	IVA

A

	N0	N1	N2
T0-2	I/II	II/IIIA	IIIA
T3	I/II/IIIA	IIIA	IIIB
T4a	IIIA	IIIB	IIIB
T4b	IVA	IVA	IVA

	TRG 0-1	TRG 2	TRG 3
T0-2N0	I	II	II
T3N0	I	II	IIIA
T0-2N1	II	II	IIIA

B

Supplementary figure 5 Kaplan-Meier survival curve of ypT stage in patients treated with nRT (A) and nCRT (B), mypN stage in patients treated with nRT (C) and nCRT (D), TRG in patients treated with nRT (E) and nCRT (F) and mypTNM stage in patients treated with nRT (G) and nCRT (H).

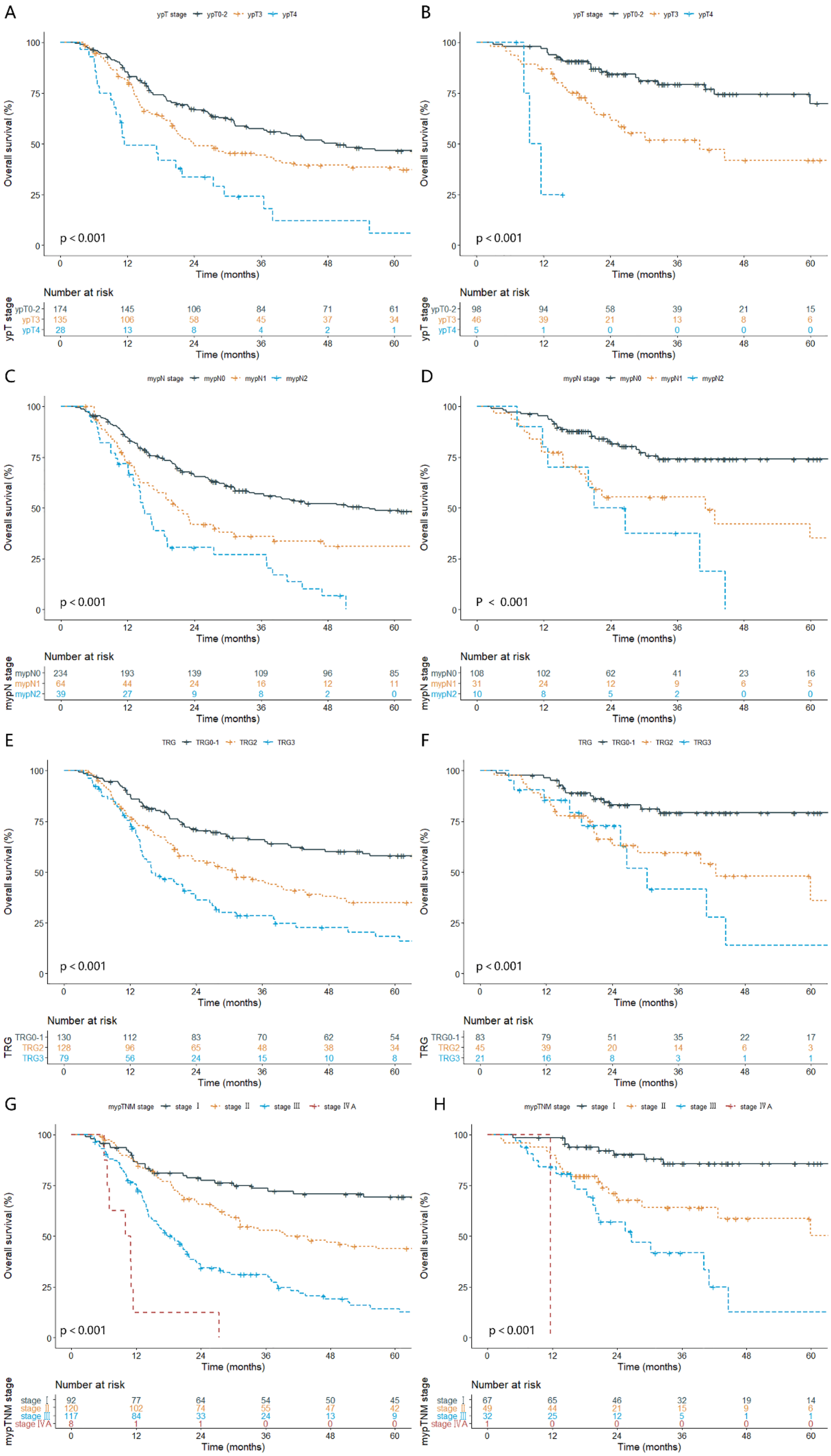
A
ypT stage ypT0-2 ypT3 ypT4
Overall survival (%)
p < 0.001
Time (months)
Number at risk
ypT stage
ypT0-2 174 145 106 84 71 61
ypT3 135 106 58 45 37 34
ypT4 28 13 8 4 2 1
B
ypT stage ypT0-2 ypT3 ypT4
Overall survival (%)
p < 0.001
Time (months)
Number at risk
ypT0-2 98 94 58 39 21 15
ypT3 46 39 21 13 8 6
ypT4 5 1 1 0 0 0
C
mypN stage mypN0 mypN1 mypN2
Overall survival (%)
p < 0.001
Time (months)
Number at risk
mypN stage
mypN0 234 193 139 109 96 85
mypN1 64 44 24 16 12 11
mypN2 39 27 9 8 2 0
D
mypN stage mypN0 mypN1 mypN2
Overall survival (%)
P < 0.001
Time (months)
Number at risk
mypN0 108 102 62 41 23 16
mypN1 31 24 12 9 6 5
mypN2 10 8 5 2 0 0
E
TRG TRG0-1 TRG2 TRG3
Overall survival (%)
p < 0.001
Time (months)
Number at risk
TRG
TRG0-1 130 112 83 70 62 54
TRG2 128 96 65 48 38 34
TRG3 79 56 24 15 10 8
F
TRG TRG0-1 TRG2 TRG3
Overall survival (%)
p < 0.001
Time (months)
Number at risk
TRG0-1 83 79 51 35 22 17
TRG2 45 39 20 14 6 3
TRG3 21 16 8 3 1 1
G
mypTNM stage stage Ⅰ stage Ⅱ stage Ⅲ stage ⅣA
Overall survival (%)
p < 0.001
Time (months)
Number at risk
mypTNM stage
stage Ⅰ 92 77 64 54 50 45
stage Ⅱ 120 102 74 55 47 42
stage Ⅲ 117 84 33 24 13 9
stage ⅣA 8 1 1 0 0 0
H
mypTNM stage stage Ⅰ stage Ⅱ stage Ⅲ stage ⅣA
Overall survival (%)
p < 0.001
Time (months)
Number at risk
stage Ⅰ 67 65 46 32 19 14
stage Ⅱ 49 44 21 15 9 6
stage Ⅲ 32 25 12 5 1 1
stage ⅣA 1 0 0 0 0 0
